# Response of microorganisms and enzymes to soil contamination with a mixture of terbuthylazine, mesotrione, and S-metolachlor

**DOI:** 10.1007/s11356-016-7919-z

**Published:** 2016-10-31

**Authors:** Agata Borowik, Jadwiga Wyszkowska, Jan Kucharski, Małgorzata Baćmaga, Monika Tomkiel

**Affiliations:** Department of Microbiology, University of Warmia and Mazury in Olsztyn, Plac Łódzki 3, 10-727 Olsztyn, Poland

**Keywords:** Microbial communities and activity, Microbiota, Enzymatic activity, Ecophysiological biodiversity, Maize

## Abstract

The research objective has been to evaluate the effect, unexplored yet, of a mixture of three active ingredients of the herbicide Lumax 537.5 SE: terbuthylazine (T), mesotrione (M), and S-metolachlor (S) on counts of soil microorganisms, structure of microbial communities, activity of soil enzymes as well as the growth and development of maize. The research was based on a pot experiment established on sandy soil with pH_KCl_ 7.0. The herbicide was applied to soil once, in the form of liquid emulsion dosed as follows: 0.67, 13.4, 26.9, 53.8, 108, 215, and 430 mg kg^−1^ of soil, converted per active substance (M + T + S). The control sample consisted of soil untreated with herbicide. The results showed that the mixture of the above active substances caused changes in values of the colony development (CD) indices of organotrophic bacteria, actinomycetes, and fungi and ecophysiological diversity (EP) indices of fungi. Changes in the ecophysiological diversity index of organotrophic bacteria and actinomycetes were small. The M + T + S mixture was a strong inhibitor of dehydrogenases, to a less degree catalase, urease, β-glucosidase, and arylsulfatase, while being a weak inhibitor of phosphatases. The actual impact was correlated with the dosage. The M + T + S mixture inhibited the growth and development of maize. The herbicide Lumax 537.5 SE should be applied strictly in line with the regime that defines its optimum dosage. Should its application adhere to the manufacturer’s instructions, the herbicide would not cause any serious disturbance in soil homeostasis. However, its excessive quantities (from 13.442 to 430.144 mg kg^−1^ DM of soil) proved to be harmful to the soil environment.

## Introduction

Intensive plant production can be destructive to the life and biodiversity of soils (Pose-Juan et al. 2015). The most recent rules governing the market implementation and approval of plant protection chemicals, in line with good experimental practice, are set out in the provisions of the Directives of the Council of the European Parliament (UE (2009) L 309) and the European and Mediterranean Plant Protection Organization [*GEP—Good Experimental Practice*] (EPPO 2016). Plant protection preparations are chemical compounds or mixtures of chemical compounds containing one or several active ingredients with mutually complementary properties but with different mechanisms of action (EPPO 2016). On the one hand, their application is exceptionally beneficial for agriculture; on the other hand, it is essential to realize what threats they can pose (Włodarczyk 2014; Jones et al. 2011; Kaczmarek et al. 2012). Exceeding manufacturer-recommended doses entails changes in the growth and development of microbial assemblages (Bello et al. 2013), plants (Snarska and Konecki 2010; Tandon et al. 2012), animals (Bro et al. 2016), and people (Nikoloff et al. 2013). Excessive use of plant protection chemicals to control pathogens and weeds can change significantly sensitivity, i.e., practical resistance. Many authors (Wyszkowska and Kucharski 2004; Kucharski and Wyszkowska 2008; Diez and Barrado 2010; Pérez-Bárcena et al. 2014; Siles et al. 2014; Lone et al. 2014; Peña et al. 2015; Kucharski et al. 2016) have demonstrated experimentally that only a small percentage of applied doses of pesticides is engaged in combating target organisms, whereas the remaining amounts permeate into the soil environment, water, air, and living organisms. The persistence of plant protection chemicals depends on the climatic conditions as well as the physical and chemical properties of soil, and in particular on the soil content of organic matter, which limits the transport of active ingredients of herbicides to water (Delgado-Moreno and Peña 2009). A low content of organic matter in degraded soils and fluctuations in moisture, temperature, precipitation, or pH are the factors that can strengthen the impact of herbicidal active substances on the structure and life of soil microbial communities (Soltani et al. 2006; Bello et al. 2013).

Plant protection preparations can produce various effects on microorganisms. As well as being toxic to some microorganisms (Martins et al. 2011; Nikoloff et al. 2013; Baćmaga et al. 2015), they are an excellent source of carbon and energy for others, which is why microorganisms play an important role in bioremediation of soils contaminated with pesticides (Delgado-Moreno and Peña 2009; Idziak and Woźnica 2008). The main source of soil enzymes are soil microorganisms and plant roots; hence, the effect of herbicides on these organisms will invariably influence the enzymatic activity of soil (Jastrzębska and Kucharski 2007; Kucharski and Wyszkowska 2008; Martins et al. 2011; Kucharski et al. 2016). While the effects produced by many herbicides have been thoroughly elucidated (Crouzet et al. 2010), we still lack information regarding the influence of the herbicide Lumax 537.5 SE on soil microorganisms and enzymes. This preparation contains three active substances: terbuthylazine, mesotrione, and S-metolachlor. Although there are studies revealing the effect of each of these substances alone on soil characteristics (Clark and Goolsby 2000; Blanchoud et al. 2007; Delgado-Moreno and Peña, 2009), no reports are available on the influence of all the three ingredients applied simultaneously in a mixture.

The substances found composing the herbicide Lumax 537.5 SE undergo chemical and microbiological degradation in the environment, and the half-life of these substances varies from a few days to about 2 months (O’Connell et al. 1998). The average half-life of terbuthylazine ranges from 11 to 35 days (Navarro et al. 2009), that of mesotrione from 6 to 34 days (Crouzet et al. 2010), and that of S-metolachlor from 24 days at a temperature of 35 °C to 65 days at 10 °C (Long et al. 2014).

Information on the impact of herbicides on the soil’s biological activity, herbicidal effectiveness, or crop yields is extremely helpful in developing crop management strategies, although changes that might be expected in field conditions should be first observed during specially designed laboratory or greenhouse experiments under controlled conditions.

Side effects of pesticides, including herbicides, are a problem that needs to be discussed, particularly in the time of their increasing use in the EU countries (Tejada 2009; Crouzet et al. 2010). The available literature lacks reports on a combined application of terbuthylazine, mesotrione, and S-metolachlor on the soil microbiome. A study was therefore undertaken to assess the interaction of these three active substances contained in Lumax 537.5 SE on the biological life of soil. In order to achieve a reliable assessment of the impact of this herbicide on the biological activity of soil, changes in the soil stability were traced over a period of time and the direction of the impact (inhibition or stimulation) produced by the preparation was determined. In addition, the influence of the mixture of terbuthylazine, mesotrione, and S-metolachlor on the growth and development of maize was evaluated. These complex investigations generated the data implicating what doses could disturb the biochemical processes in soil. Knowledge of the influence of herbicides and their metabolites on these parameters can be used for biomonitoring of the soil environment.

The principal aim of our study was therefore to identify the response of soil microorganisms and enzymes, i.e., dehydrogenases, urease, alkaline phosphatase, acid phosphatase, β-glucosidase, and arylsulfatase, to a mixture of three active substances found in the herbicide Lumax 537.5 SE.

## Research material and methods

### Soil

The first step was to select soil for our studies, to which aim analyses were made of arable soils at the Research Station in Tomaszkowo, which belongs to the University of Warmia and Mazury in Olsztyn (NE Poland, 53.7167° N, 20.4167° E). The Research Station in Tomaszkowo lies in the Olsztyn Lake District, which is part of the Masurian Lake District. The dominant soil types are the ones classified into Order 3: Brown earths type 3.1; eutrophic brown soils type 3.1. Eutric Cambisols. In respect of the grain size distribution according to the IUSS Working Group WRB: World Reference Base for Soil Resources (2014), the soil selected for our research belongs to subtype 3.1.1 Endocalcaric Cambisols. Soil samples were obtained from the arable humic horizon (0–20 cm depth). Regarding the particle size composition, this soil represented sandy loam. The basic characteristics of this soil are presented in Table 1.

**Table 1:** General characteristics of experimental soil

Sand	Silt	Clay	C_org_	N_tot_	HAC	EBC	CEC	BS	pH_KCl_
*Ø* μm									
50–2000	2–50	<2							
g kg^−1^					mM(+) kg^−1^			%	
720	210	70	7.05	0.86	8.00	111.00	119.00	93.27	7.00

*HAC* hydrolytic acidity, *EBC* exchangeable base cations, *CEC* sorption capacity, *BS* base saturation, *C*_*org*_ organic carbon content, *N*_*tot*_ total nitrogen content

### Herbicide

Three active ingredients were tested: terbuthylazine (T), mesotrione (M), and S-metolachlor (S), which are contained in the preparation Lumax 537.5 SE, a herbicide made by Syngenta Crop Protection. Both top-dressing and foliar application of this preparation are possible. In line with the classification by the Herbicide Resistance Action Committee (HRAC) 2016 (CASAFE 2011 vs 2012; HRAC 2016), Lumax 537.5 SE is a herbicide used for control of monocotyledonous (especially Panicoideae) and dicotylodenous weeds, prior to or immediately after emergence of maize, until the third phase of the crop. The manufacturer’s recommended doses range from 3.5 to 4.0 dm^3^ ha^−1^, which equal 1.17 to 1.33 mm^3^ kg^−1^. As this was a greenhouse experiment, set up in 3.5-dm^3^ pots, doses of the herbicide were expressed in quantities per 1 kg of soil. For this purpose, it was assumed that an area of 1 ha holds 3,000,000 kg of soil in a layer of 0 to 20 cm in depth and at the soil density is 1.5 g cm^3^. Lumax 537.5 SE was applied to soil in the form of aqueous suspension. The innovative feature of the herbicide is that it integrates the action of three active substances: terbuthylazine 6-chloro-*N*-(1,1-dimethylethyl)-*N*′-ethyl-1,3,5-triazine-2,4-diamine, mesotrione: 2-[4-(methylsulfony)-2-nitrobenzoyl]-cyclohexane 1,3-dion, and S-metolachlor: (*S*)-2-chloro-*N*-(2-ethyl-6-methylphenyl)-*N*-(2-methoxy-1-methylethyl) acetamide. In addition, the herbicide contains alpha-(tris(1-phenylethyl)phenyl)-omega-hydroxy poly(oxy-1,2-ethanediyl), dioctyl sodium sulfosuccinate, and propane 1,2-diol. One cubic decimetre of the herbicide contains 187.5 g of terbuthylazine, 37.5 g of mesotrione, and 312.5 g of S-metolachlor. These substances differ in their degradability (Table 2). The predicted environmental concentrations (PEC) of the active substances in soil are presented in Table 2.

**Table 2:** Predicted environmental concentrations (PEC) of T + M + S in soil (mg kg^−1^)

Active ingredient								
Terbuthylazine (T)			Mesotrione (M)			S-metolachlor (S)		
Dose	Analysis day		Dose	Analysis day		Dose	Analysis day	
	30	60		30	60		30	60
mg kg^−1^								
0.234	0.093	0.037	0.047	0.003	0.000	0.3906	0.145	0.054
4.688	1.853	0.732	0.942	0.015	0.000	7.812	2.902	1.078
9.376	3.706	1.464	1.884	0.029	0.000	15.624	5.804	2.156
18.752	7.411	2.929	3.768	0.059	0.001	31.248	11.609	4.313
37.504	14.822	5.858	7.536	0.118	0.002	62.496	23.217	8.625
75.008	29.644	11.716	15.072	0.236	0.004	124.992	46.434	17.250
150.016	59.289	23.432	30.144	0.471	0.007	249.984	92.869	34.501

### Research protocol

Having selected the herbicide and the soil, and once the soil properties were determined (Table 1), the subsequent research stage was carried out in a greenhouse, under controlled conditions. Before the trials began, sandy loam soil was passed through a sieve with the mesh size of 5 mm. Three-kilo batches of soil were thoroughly mixed with previously prepared water suspension of Lumax 537.5 SE, containing a mixture of terbuthylazine, mesotrione, and S-metolachlor, and with mineral fertilizers, after which they were transferred to plastic pots. The herbicide was applied to soil once, in the form of water emulsion, in the following doses (converted to the active substances in mg kg^−1^ of soil): 0.672 (manufacturer’s recommended dose), 13.442, 26.884, 53.768, 107.536, 215.072, and 430.144. The control was composed of soil without any application of the herbicide. Each series of the experiment with the same dose was replicated four times. Higher doses of the mixture of terbuthylazine, mesotrione, and S-metolachlor were incorporated into soil to assess possible threats arising from an incidental and uncontrolled penetration of the above substances into the soil environment. Doses of mineral fertilizers were adjusted to the nutritional requirements of maize and reached (converted to pure component per mg kg^−1^ of soil) the following: N—100 [CO(NH_2_)]_2_, P—44 [KH_2_PO_4_], K—100 [KH_2_PO_4_ + KCl], Mg—25 [MgSO_4_·7H_2_O], Cu—5 [CuSO_4_·5H_2_O], Zn—5 [ZnCl_2_], Mn—5 [MnCl_2_·4H_2_O], Mo—2.5 [Na_2_MoO_4_·2H_2_O] and B—0.33 [H_3_BO_4_]. The soil moisture content was adjusted with deionized water to the level of 50 % of water capillary capacity. Next, maize of the variety LG 32.58 FAO 250 was sown in pots (five plants per pot). The soil moisture content was constantly monitored and kept constant throughout the experiment. On days 30 and 60, soil samples were taken from each pot with a given dose of the mixture of terbuthylazine, mesotrione, and S-metolachlor, producing an aggregated sample weighing 500 g, which was submitted to microbiological (five replications for each sample) and biochemical (three replications per sample) determinations.

### Soil microorganisms

Twice during the experiment, i.e., on days 30 and 60, soil samples with a particular dose of the terbuthylazine, mesotrione, and S-metolachlor mixture underwent microbiological determinations, in five replications, such as counts of oligotrophic and endospore-forming oligotrophic bacteria, on 100-fold diluted organotrophic bacteria—on Bunta and Roviry, *Azotobacter* spp. bacteria—on Fenglerowa, actinomycetes—on Küster and Williams medium supplemented with nystatin and actidione, and fungi—on the glucose-peptide medium with rose bengal and aureomycin (Martin 1950; Fenglerowa 1965; Parkinson et al. 1971; Alexander 1973; Onta and Hattori Onta and Hattori 1983). All microorganisms were grown at 28 °C.

The impact of terbuthylazine, mesotrione, and S-metolachlor on the structure of communities of organotrophic bacteria, actinomycetes, or fungi and on their ecophysiological diversity was explored. To this aim, on days 30 and 60 of the experiment, appropriate dilutions of the soil solution suspension were inoculated onto Petri dishes, in five parallel replicates, and then incubated at a temperature of 28 °C. For ten consecutive days, grown colonies of microorganisms were counted daily and, based on the attained growth dynamics, conclusions were drawn with respect to the microbiological diversity of the soil. The observations were supported by the colony development index CD (Sarathchandra et al. 1997) and the ecophysiological diversity index EP (De Leij et al. 1993) described in manuscripts by Baćmaga et al. (2015) and Borowik and Wyszkowska (2016).

The values of the CD and EP indices depended on both a dose of the herbicide and the maize’s growing time. The highest values of the CD index were achieved by fungi. An increase in the CD index suggests that the proportion of rapidly growing microorganisms (r-strategists) is on the increase while that of slowly growing microorganisms (K-strategists) is decreasing. The CD index ranges from 10 to 100. The CD index reaches 100 when all colonies of microorganisms isolated from soil have grown after 24 h. The CD index value of 10 means that all colonies have grown on day 10. The CD value of 29 indicates the uniform growth of microbial colonies daily over 10 days.

The EP index ranges from 0 to 1 and informs about the rate at which a colony of microorganisms isolated from soil appears. If the value of this index is 1, it means that the same number of colonies appears on each day over 10 days (De Leij et al. 1993).

### Soil enzymes

The activity of enzymes, analogously to the counts of microorganisms, was determined in soil samples obtained on days 30 and 60 of the experiment. The determinations were performed in three replicates for each combination. The activity of the following enzymes was tested: dehydrogenases (EC 1.1)—with the Lenhard method modified by Öhlinger (1996), catalase (EC 1.11.1.6), urease (EC 3.5.1.5), arylsulfatase (EC 3.1.6.1), β-glucosidase (EC 3.2.1.21), acid phosphatase (EC 3.1.3.2), and alkaline phosphatase (EC 3.1.3.1)—according to Alef and Nannipieri (1998).

The following substrates were used: 2,3,5-triphenyl tetrazolium chloride TTC for dehydrogenases, hydrogen peroxide for catalase, 4-nitrophenyl phosphate disodium PNPNa for phosphatases, urea for urease, *p*-nitrophenyl-β-d-glucopyranoside PNG for β-glucosidase, and potassium 4-nitrophenyl sulfate—PNS for arylsulfatase. All substrates were purchased from Sigma-Aldrich. The activity of the soil enzymes was expressed in the following units, in 1 kg DM of soil h^−1^: dehydrogenase in micromolar of triphenyl formazan (TPF); catalase—molar O_2_; urease—millimolar N-NH_4_; and acid phosphatase, alkaline phosphatase, β-glucosidase, and arylsulfatase—millimolar of *p*-nitrophenol (PNP). Determinations of the activity of all enzymes except catalase were made on a Perkin-Elmer Lambda 25 spectrophotometer (MA, USA).

### Evaluation of the growth and development of maize

Maize was harvested on day 60 of the experiment, in the early heading phase (BBCH 51). The aerial parts of the plants were dried at 65 °C; afterwards, they were weighed and results were processed statistically. The dry matter yield of maize was expressed in grams per pot.

### Physicochemical properties of soil

Before the experiment was started, soil samples were submitted to the following determinations: textural composition with a Mastersizer laser particle size analyzer produced by Malvern (Worcestershire, UK), reaction (pH) by potentiometric in an aqueous solution of KCl at the concentration of 1 mol dm^3^ (ISO 10390, 2005), hydrolytic acidity (HAC) and exchangeable base cations (EBC) by the Kappen method (Klute 1996), content of total nitrogen according to the method by Kjeldahl (ISO 11261: 1995), and organic carbon (C_org_) content by the Tiurin method (Nelson and Sommers 1996). Based on the HAC and EBC values, the cation exchange capacity (CEC) and base saturation (BS) of the soil were computed. The following equations were applied: CEC = EBC + HAC; BS = (EBC/CEC) · 100.

### Determination of the indices measuring the effect of a mixture of the herbicidal active substances

Based on the counts of microorganisms and activity of soil enzymes, the value of the impact index of a mixture of terbuthylazine, mesotrione, and S-metolachlor was derived from the following formula:

$$ {I}_{\mathrm{I}/\mathrm{S}}=\frac{P_b}{P_k}-1 $$

where *I*_I/S_ is the index of the impact (inhibition or stimulation) of the herbicide, *P*_*b*_ is counts of microorganisms or activity of enzymes in the soil polluted with the herbicide, and *P*_*k*_ is counts of microorganisms or activity of enzymes in the soil not polluted with the herbicide. If *I*_I/S_ = 1, it indicates 100 % stimulation of the mixture of terbuthylazine, mesotrione, and S-metolachlor on a given parameter of the soil microbiome; *I*_I/S_ = −1 indicates 100 % corresponds to inhibition; *I*_I/S_ = 0 indicates absence of the impact.

### Statistical analyses

In line with the principles of rational deduction, the research results were statistically analyzed with the help of the software program STATISTICA 12.0 (Statsoft, Inc., Statistica 2015). For an easier interpretation of the effects of the mixture of terbuthylazine, mesotrione, and S-metolachlor on the soil microbiome, it was helpful to determine the percentage contribution of particular independent variables to the shaping of dependent variables. To this aim, we used an analysis of the measure of an effect *η* ^2^ made by analysis of variance (ANOVA). Homogenous groups were distinguished by Tukey’s test, at *P* = 0.05. Values of Pearson’s simple correlation coefficients were calculated between the dependent and independent variables. The results were submitted to principal component analysis (PCA) interpretation. PCA is an algorithm based on matrix calculations. It consists of determination of primary components which are a linear combination of analyzed variables. PCA is a method of the transformation of observable primary variables into new, mutually orthogonal variables, i.e., principal components. It is possible to establish as many principal components as there were primary variables. PCA allows the user to identify these initial variables which have large influence on the shape of individual principal components. PCA consists in observations of a set of data in a dimensional space, in which the highest variability is presented by the first two analyzed factors, which in our case were doses of the M + T + S mixture and day of analysis. If the vectors representing primary variables reach close to the edges of a circle with the radius equal to 1, then they are very well represented by the first two principal components that create a set of coordinates. If the angle between the vectors is small, it indicates high correlation between these variables. Analysis of variance was employed to assess distances between clusters. The distance between the clusters was measured with Ward’s method, using Euclidean metrics. The varied effects of the mixture of the active substances contained in the herbicide on the soil microbiome were illustrated by the impact (inhibition or stimulation) of the herbicide.

## Results

### Soil microorganisms

In accord with the proposed hypothesis, counts of all microorganisms depended on both the mixture of terbuthylazine, mesotrione, and S-metolachlor and the duration of the experiment (Table 3). The statistical analysis of the contribution of all the factors to the detected variability *η* ^2^ showed that a dose of the preparation decided in the range of 17 % (oligotrophic bacteria) to 56 % (fungi) on the counts of microorganisms, while the date of the analysis affected microbial counts from 10 % (fungi) to 69 % (oligotrophic bacteria). In the unpolluted soil samples (Table 4), counts of organotrophic bacteria, *Azotobacter*, actinomycetes, and fungi, in contrast to endospore-forming oligotrophic bacteria, were significantly higher in the BBH 51 maize development phase (day 60 of the plants’ growth) than in the BBCH 31 phase (day 30). Counts of oligotrophic bacteria were comparable in both maize development phases.

**Table 3:** Percent of the observed variability *η* ^2^ in soil contaminated with the mixture T + M + S

Variable factors	Microorganisms					
	Olig	Olig_p_	Az	Org	Act	Fun
T + M + S dose	17.104	45.450	19.502	47.439	30.658	55.534
Analysis day	68.843	26.837	61.041	21.789	31.643	10.074
Dose × time	11.726	26.443	12.600	24.942	25.249	30.985
Error	2.327	1.270	6.857	5.830	12.450	3.408

*Org* organotrophic bacteria, *Act* actinomycetes, *Fun* fungi, *Olig* oligotrophic bacteria, *Olig*_*p*_ oligotrophic spores, *Az* bacteria of the genus *Azotobacter*, *T* terbuthylazine, *M* mesotrione, *S* S-metolachlor

**Table 4:** Counts of microorganisms per 1 kg DM of soil unpolluted with M + T + S

Analysis day	Olig 10^9^	Oligp 10^8^	Az 10^3^	Org 10^9^	Act 10^9^	Fun 10^5^
30	13.549^a^±1.075	5.643^a^±0.854	14.615^b^±0.386	12.119^b^±1.431	9.564^b^±0.881	22.220^b^±4.807
60	13.749^a^±3.231	4.322^b^±0.943	55.06^a^±0.696	28.474^a^±8.842	34.609^a^±6.500	56.619^a^±11.947
Average	13.649	4.983	34.838	20.297	22.087	39.420

The same letters in the columns indicate homogeneous groups

*T* terbuthylazine, *M* mesotrione, *S* S-metolachlor

**Fig. 1:**
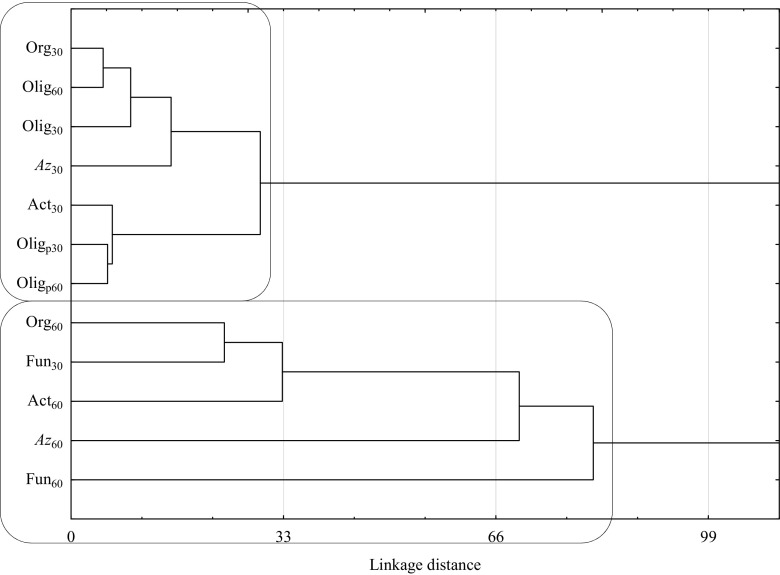
Similar response of microorganisms in soil contaminated with T + M + S. *Org* organotrophic bacteria, *Act* actinomycetes, *Fun* fungi, *Olig* oligorophic bacteria, *Olig*_*p*_ oligorophic bacteria with spore, *Az* bacteria of the genus *Azotobacter*. Date of analysis (days): *30* 30 days, *60* 60 days

The excessive doses of the mixture of terbuthylazine, mesotrione, and S-metolachlor applied to soil interfered with the soil’s microbiological equilibrium, measured by the counts of oligotrophic bacteria, endospore-forming oligotrophic bacteria, *Azotobacter* spp., organotrophic bacteria, actinomycetes, and fungi. In our experiment, the mixture of the three active substances produced a significant negative impact on the soil microbiome (Table 5). This conclusion is supported by the following finding: the vast majority of the herbicide-polluted soil samples produced negative impact index (*I*_I/S_) values regarding the effect of the herbicide on microorganisms. Endospore-forming oligotrophic bacteria were an exception. The response of microorganisms to the active ingredients of Lumax 537.5 SE was stronger on day 60 than on day 30 of the experiment. The lowest counts of all microorganisms were observed in soils added the dose of 430.144 mg T + M + S kg^−1^. The severe stress induced by such a large dose of the herbicide decreased the count of oligotrophic bacteria by 24 %, fungi by 55 %, actinomycetes by 79 %, and *Azotobacter* spp. by 96 % on day 60. The differentiated response of microorganisms to the presence of Lumax 537.5 SE in soil, depending on the duration of the preparation’s impact, is confirmed by the cluster analysis (CA) carried out according to Ward’s method (Fig. 1). Two groups of microorganisms similar in response to the soil pollution with terbuthylazine, mesotrione, and S-metolachlor can be distinguished from the achieved dendrogram. There are some sub-groups seen within the two major groups, which justifies the claim that organotrophic bacteria, *Azotobacter* spp. bacteria, and actinomycetes responded differently to the soil contamination on the second and on the first dates of analyses. It is worth emphasizing that the greatest similarity in the response to the pollution with the herbicide occurred between organotrophic bacteria on day 30 and oligotrophic bacteria on day 60.

**Table 5:** Effect of Lumax 537.5 SE on the development of microorganisms in soil, expressed by the index of inhibition or stimulation (*I*_I/S_)

Dose T + M + S (mg kg^−1^)	Microorganisms					
	Olig	Olig_p_	Az	Org	Act	Fun
Analysis day 30						
0.6721	−0.102^b^±0.040	0.152^c^±0.099	−0.135^b^±0.035	−0.105^a^±0.043	−0.147^a^±0.044	−0.101^a^±0.064
13.442	−0.164^d^±0.052	0.292^a^±0.050	−0.005^a^±0.005	−0.191^b^±0.025	−0.240^b^±0.063	−0.152^b^±0.069
26.884	−0.247^e^±0.030	0.181^c^±0.085	−0.522^d^±0.246	−0.208^b^±0.038	−0.228^b^±0.048	−0.223^cd^±0.069
53.768	−0.137^c^±0.045	0.042^e^±0.018	−0.674^e^±0.238	−0.216^b^±0.043	−0.266^b^±0.060	−0.199^c^±0.080
107.536	−0.114^bc^±0.047	−0.151^f^±0.071	−0.888^f^±0.080	−0.310^c^±0.092	−0.418^c^±0.129	−0.103^a^±0.044
215.072	−0.053^a^±0.037	0.104^d^±0.078	−0.746^e^±0.267	−0.346^c^±0.039	−0.504^d^±0.086	−0.220^cd^±0.066
430.144	−0.029^a^±0.027	0.246^b^±0.171	−0.339^c^±0.088	−0.440^d^±0.059	−0.622^e^±0.077	−0.250^d^±0.080
Average	−0.121	0.124	−0.473	−0.259	−0.346	−0.178
Analysis day 60						
0.6721	−0.075^a^±0.028	0.312^c^±0.065	0.228^a^±0.131	0.389^a^±0.126	−0.048^a^±0.014	0.052^a^±0.030
13.442	−0.194^b^±0.020	0.429^a^±0.062	−0.506^b^±0.058	−0.275^c^±0.066	−0.454^b^±0.027	−0.216^b^±0.070
26.884	−0.283^cd^±0.030	0.374^b^±0.093	−0.836^c^±0.031	−0.278^cd^±0.061	−0.457^b^±0.060	−0.249^b^±0.078
53.768	−0.373^e^±0.027	0.230^d^±0.037	−0.959^c^±0.028	−0.282^cd^±0.036	−0.789^c^±0.020	−0.313^c^±0.042
107.536	−0.446^f^±0.032	0.064^e^±0.055	−0.966^c^±0.046	−0.333^d^±0.033	−0.855^c^±0.015	−0.431^d^±0.064
215.072	−0.318^d^±0.020	−0.190^f^±0.075	−0.978^c^±0.020	−0.333^d^±0.045	−0.798^c^±0.011	−0.469^d^±0.093
430.144	−0.238^bc^±0.032	−0.412^g^±0.113	−0.959^c^±0.021	−0.029^b^±0.040	−0.793^c^±0.025	−0.547^e^±0.062
average	−0.275	0.115	−0.711	−0.163	−0.599	−0.310

The same letters in the columns indicate homogeneous groups

*Org* organotrophic bacteria, *Act* actinomycetes, *Fun* fungi, *Olig* oligotrophic bacteria, *Olig*_*p*_ oligotrophic spores, *Az* bacteria of the genus *Azotobacter*, *T* terbuthylazine, *M* mesotrione, *S* S-metolachlor

**Fig. 2:**
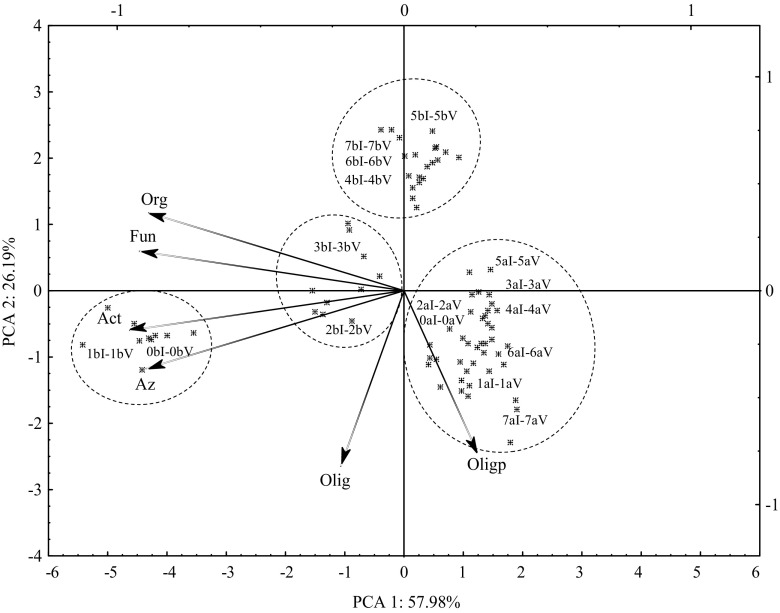
Counts of microorganisms in soil contaminated with T + M + S represented with the PCA. *Org* organotrophic bacteria, *Act* actinomycetes, *Fun* fungi, *Olig* oligotrophic bacteria, *Olig*_*p*_ oligorophic bacteria with spore, *Az* bacteria of the genus *Azotobacter*. Dose of herbicide in milligrams per kilogram DM of soil: *0* 0 (control), *1* 0.672, *2* 13.442, *3* 26.884, *4* 53.768, *5* 107.536, *6* 215.072, *7* 430.144. Analysis day: *a* 30, *b* 60; number of repetitions: *I*–*V*

The effect of the mixture of terbuthylazine, mesotrione, and S-metolachlor on the consortium of bacteria can be summarized by presenting the dispersion of counts of individual groups of microorganisms in a set of the two first principal components (Fig. 2). The first principal component carries 57.98 % of the total variance of the variables describing the abundance of microorganisms. Along this axis, there are vectors characterized by the high negative fit representing primary variables of organotrophic bacteria, *Azotobacter* spp. bacteria, and fungi, which were highly significantly positively correlated. The vertical axis, along which there are vectors corresponding to oligotrophic bacteria and endospore-forming oligotrophic bacteria, explains 26.19 % of the total variance of the variables. The projection of cases on the plane of factors proves that the highest growth of bacteria *Azotobacter*, organotrophic bacteria, actinomycetes, and fungi was noted in non-polluted soil in the second date of analyses and in the soil supplemented with the dose of Lumax 537.5 SE recommended by the producer. T + M + S did not cause a decrease in the count of endospore-forming oligotrophic bacteria. Our analysis of the distribution of particular cases, representing the counts of microorganisms and the percent of the observed variability of *η* ^2^, shows that the soil microbiome was determined by both a dose of the herbicide as well as the duration of the experiment.

The mixture of terbuthylazine, mesotrione, and S-metolachlor, by altering the living conditions for soil microbiota, led to the structural differentiation of microorganisms (Tables 6 and 7). The variation of organotrophic bacteria (days 30 and 60) and actinomycetes (day 60) was higher in non-polluted than in polluted soils, whereas fungi (day 30) and actinomycetes (day 30) were higher in non-contaminated than in contaminated soils (Table 7).

**Table 6:** Colony development index (CD) in soil contaminated with the mixture T + M + S

Dose T + M + S (mg kg^−1^)	Microorganisms					
	Organotrophic bacteria		Actinomycetes		Fungi	
	Analysis day					
	30	60	30	60	30	60
0	36.816^a^±1.712	44.555^a^±10.419	29.635^a^±1.052	34.739^a^±1.727	40.159^abc^±5.390	45.022^abc^±4.092
0.6721	32.512^c^±2.940	40.522^c^±4.432	24.997^b^±0.351	25.864^b^±0.839	35.078^bc^±4.376	46.932^bc^±2.756
13.442	33.235^bc^±1.646	34.376^bc^±1.869	22.806^bcd^±1.031	29.167^bcd^±2.508	37.481^bc^±1.441	47.288^bc^±2.662
26.884	31.853^c^±1.056	38.785^c^±2.483	24.017^bc^±1.494	29.230^bc^±1.442	34.509^c^±1.671	41.524^c^±6.454
53.768	35.397^ab^±3.292	33.674^ab^±3.735	22.916^bcd^±1.263	23.784^bcd^±5.295	40.528^abc^±4.376	52.896^abc^±4.606
107.536	33.363^bc^±2.886	27.572^bc^±1.001	22.864^bcd^±3.383	26.860^bcd^±5.534	41.077^ab^±2.026	56.898^ab^±6.312
215.072	32.544^c^±1.131	37.429^c^±4.583	20.924^d^±4.542	24.166^d^±4.758	44.485^a^±6.511	47.131^a^±4.379
430.144	31.123^c^±2.183	26.604^c^±1.348	22.022^cd^±2.205	21.269^cd^±6.839	41.000^ab^±5.014	47.458^ab^±3.994
Average	33.355	35.440	23.773	26.885	39.290	48.144

The same letters in the columns indicate homogeneous groups

T terbuthylazine, M mesotrione, S S-metolachlor

**Table 7:** Ecophysiological biodiversity index (EP) in soil contaminated with the mixture T + M + S

Dose T + M + S (mg kg^−1^)	Microorganisms					
	Organotrophic bacteria		Actinomycetes		Fungi	
	Analysis day					
	30	60	30	60	30	60
0	0.897^ab^±0.038	0.758^d^±0.085	0.940^a^±0.039	0.818^de^±0.056	0.555^a^±0.100	0.562^ab^±0.075
0.6721	0.887^b^±0.049	0.781^cd^±0.043	0.862^b^±0.080	0.877^ab^±0.064	0.561^a^±0.102	0.549^ab^±0.076
13.442	0.896^ab^±0.038	0.808^bcd^±0.053	0.859^b^±0.060	0.841^bcde^±0.048	0.412^abc^±0.082	0.581^a^±0.059
26.884	0.930^a^±0.050	0.773^d^±0.047	0.864^b^±0.033	0.811^e^±0.038	0.353^c^±0.066	0.602^a^±0.057
53.768	0.894^ab^±0.015	0.881^a^±0.039	0.863^b^±0.061	0.829^cde^±0.098	0.388^bc^±0.171	0.566^ab^±0.042
107.536	0.889^b^±0.059	0.838^ab^±0.025	0.869^b^±0.053	0.858^bcd^±0.087	0.399^bc^±0.054	0.578^a^±0.092
215.072	0.871^b^±0.060	0.830^bc^±0.037	0.875^b^±0.061	0.906^a^±0.058	0.525^ab^±0.099	0.461^b^±0.157
430.144	0.896^ab^±0.022	0.800^bcd^±0.037	0.887^b^±0.029	0.870^abc^±0.114	0.370^bc^±0.0193	0.327^c^±0.097
Average	0.895	0.808	0.877	0.851	0.445	0.528
*r*	−0.255	0.165	0.047	0.537	−0.289	−0.948

The same letters in the columns indicate homogeneous groups

*T* terbuthylazine, *M* mesotrione, *S* S-metolachlor

The colony development (CD) index of organotrophic bacteria ranged from 26.604 (430.144 mg T + M + S kg^−1^ DM of soil, 60 days) to 44.555 (0 mg T + M + S kg^−1^ DM of soil, 60 days), that of actinomycetes from 20.924 (215.072 mg T + M + S kg^−1^ DM of soil, 30 days) to 34.739 (0 mg T + M + S kg^−1^ DM of soil, 60 days), and that of fungi from 34.509 (26.884 mg T + M + S kg^−1^ DM of soil, 30 days) to 56.898 (107.536 mg T + M + S kg^−1^ DM of soil, 60 days). Leaving aside the question of the doses of Lumax 573.5 SE, higher mean values of the CD index for organotrophic bacteria, actinomycetes, and fungi were noted on day 60 than on day 30 of the experiment. Among all the groups of microorganisms, fungal colonies grew most rapidly (the average CD value ranged from 39.290 to 48.144), while those of actinomycetes were observed to grow the most slowly (the mean CD value from 23.773 to 26.885).

The variation in counts and groups of soil microorganisms is illustrated by the ecophysiological diversity index (EP). The lowest EP values were determined for fungi, and higher ones for organotrophic bacteria and actinomycetes (Table 7). The EP values for organotrophic bacteria and actinomycetes only slightly depended on the degree of soil contamination with the herbicide, although the higher doses of T + M + S significantly depressed the EP index value of fungi. The highest average EP index value was noted for organotrophic bacteria (0.895) on day 30 of the experiment, while the lowest one was computed for fungi (0.445) on the same day. Intermediate values of EP were found for actinomycetes (from 0.851 to 0.877).

### Activity of soil enzymes

The percent of observed variability of the activity of soil enzymes in this experiment, like the counts of microorganisms, was most strongly dependent on a dose of the mixture of terbuthylazine, mesotrione, and S-metolachlor, e.g., acid phosphatase in 27 %, catalase in 43 %, arylsulfatase in 52 %, alkaline phosphatase in 57 %, dehydrogenases in 83 %, urease in 89 %, and β-glucosidase in 92 % (Table 8). In soil unpolluted with the T + B + S mixture, the activity of just one enzyme, namely alkaline phosphatase, was significantly higher on day 30 of the experiment than on day 60 (Table 9). The activity of the other six enzymes was higher on day 60.

**Table 8:** Percent of the observed variability *η* ^2^ in soil contaminated with the mixture T + M + S

Variable factors		Enzymes					
	Deh	Cat	Ure	Pac	Pal	Aryl	Glu
T + M + S dose	83.085	42.539	88.649	26.995	57.002	51.917	91.927
Analysis day	4.481	0.174	0.023	62.427	37.706	2.142	0.295
Dose × time	12.377	56.520	11.059	10.213	1.053	44.481	7.199
Error	0.057	0.767	0.269	0.365	4.238	1.459	0.578

*Deh* dehydrogenases, *Cat* catalase, *Ure* urease, *Pac* acid phosphatase, *Pal* alkaline phosphatase, *Aryl* arylsulfatase, *Glu* β-glucosidase, *T* terbuthylazine, *M* mesotrione, *S* S-metolachlor

**Table 9:** Activity of enzymes per 1 kg DM of soil unpolluted with M + T + S

Analysis day	Dehydrogenases	Catalase	Urease	Acid phosphatase	Alkaline phosphatase	Arylsulfatase	β-Glucosidase
	μM TFF	M O_2_	mM N-NH_4_	mM PNP			
30	14.154^b^±0.743	0.212^b^±0.000	0.607^b^±0.060	1.654^b^±0.100	2.980^a^±0.080	0.269^b^±0.015	0.285^b^±0.042
60	30.176^a^±0.632	0.340^a^±0.006	0.781^a^±0.036	3.121^a^±0.119	2.682^b^±0.078	0.438^a^±0.010	0.466^a^±0.009
Average	22.165	0.276	0.694	2.388	2.831	0.354	0.376

The same letters in the columns indicate homogeneous groups

*T* terbuthylazine, *M* mesotrione, *S* S-metolachlor

It is evident from the analysis of changes in the biochemical properties of soil that dehydrogenases were the most sensitive enzymes, regardless of the soil incubation period (Table 10). Even a low dose of T + M + S such as 13.442 mg kg^−1^ DM of soil decreased the activity of this enzyme by over 50 % relative to the control sample. A decrease in the activity of enzymes in excess of 50 % was induced by the dose of 53.768 mg T + M + S in regard to urease on both dates of analysis and catalase on day 60, as well as the dose of 215.072 mg T + M + S with respect to arylsulfatase and β-glucosidase on day 60. The tested substances produced a lasting inhibitory effect on the analyzed enzymes, which persisted and even intensified over time. Acid phosphatase was an exception in that its activity on day 60 of the experiment was less inhibited by the herbicide than on day 30. However, with respect to their sensitivity to the tested preparation, the enzymes can be ordered as follows: Deh > Pac > Ure > Glu > Cat > Pal > Aryl on day 30 and Deh > Cat > Ure > Glu > Aryl > Pac > Pal on day 60.

**Table 10:** Effect of Lumax 537.5 SE on soil enzymes, expressed by the index of inhibition or stimulation (*I*_I/S_)

Dose T + M + S (mg kg^−1^)	Dehydrogenases	Catalase	Urease	Acid phosphatase	Alkaline phosphatase	Arylsulfatase	β-Glucosidase
Analysis day 30							
0.6721	−0.056^a^±0.018	−0.071^d^±0.000	0.329^a^±0.035	−0.011^a^±0.006	0.114^a^±0.045	0.041^c^±0.024	0.035^a^±0.018
13.442	−0.503^b^±0.010	−0.269^g^±0.032	0.015^b^±0.007	−0.307^b^±0.035	0.007^b^±0.004	0.056^b^±0.020	−0.004^b^±0.003
26.884	−0.539^b^±0.004	0.014^c^±0.001	−0.194^c^±0.014	−0.320^bc^±0.068	−0.002^b^±0.002	0.052^b^±0.017	−0.081^c^±0.025
53.768	−0.741^c^±0.014	−0.208^f^±0.000	−0.511^f^±0.036	−0.349^c^±0.044	−0.035^c^±0.011	−0.059^e^±0.016	−0.084^c^±0.031
107.536	−0.791^c^±0.015	0.052^b^±0.004	−0.405^e^±0.014	−0.405^d^±0.015	−0.097^d^±0.028	−0.112^f^±0.039	−0.126^d^±0.047
215.072	−0.857^d^±0.003	0.033^a^±0.000	−0.402^e^±0.022	−0.409^d^±0.003	−0.155^e^±0.055	0.015^d^±0.013	−0.221^e^±0.057
430.144	−0.903^d^±0.000	−0.108^e^±0.031	−0.357^d^±0.012	−0.411^d^±0.004	−0.178^e^±0.044	0.074^a^±0.027	−0.225^e^±0.026
Average	−0.627	−0.080	−0.218	−0.316	−0.049	0.010	−0.101
Analysis day 60							
0.6721	−0.286^a^±0.005	−0.253^b^±0.000	0.288^a^±0.017	0.292^a^±0.040	0.064^a^±0.030	0.039^a^±0.010	−0.238^a^±0.008
13.442	−0.634^b^±0.019	−0.238^b^±0.033	−0.012^b^±0.007	0.125^b^±0.037	−0.022^b^±0.009	−0.128^b^±0.028	−0.275^b^±0.002
26.884	−0.672^b^±0.011	−0.779^e^±0.019	−0.273^c^±0.024	0.039^c^±0.024	−0.095^c^±0.033	−0.288^c^±0.043	−0.294^b^±0.004
53.768	−0.919^c^±0.002	−0.500^c^±0.018	−0.789^d^±0.037	−0.286^d^±0.015	−0.110^d^±0.016	−0.521^e^±0.017	−0.438^c^±0.025
107.536	−0.944^c^±0.006	−0.703^d^±0.018	−0.818^e^±0.039	−0.304^d^±0.022	−0.174^e^±0.014	−0.477^d^±0.029	−0.498^d^±0.013
215.072	−0.960^c^±0.008	−0.668^d^±0.019	−0.849^ef^±0.015	−0.434^e^±0.037	−0.204^f^±0.017	−0.628^f^±0.035	−0.506^d^±0.005
430.144	−0.981^c^±0.001	−0.132^a^±0.036	−0.598^f^±0.014	−0.423^e^±0.024	−0.249^g^±0.043	−0.612^f^±0.042	−0.517^d^±0.006
Average	−0.771	−0.468	−0.436	−0.141	−0.113	−0.373	−0.395

The same letters in the columns indicate homogeneous groups

*T* terbuthylazine, *M* mesotrione, *S* S-metolachlor

**Fig. 3:**
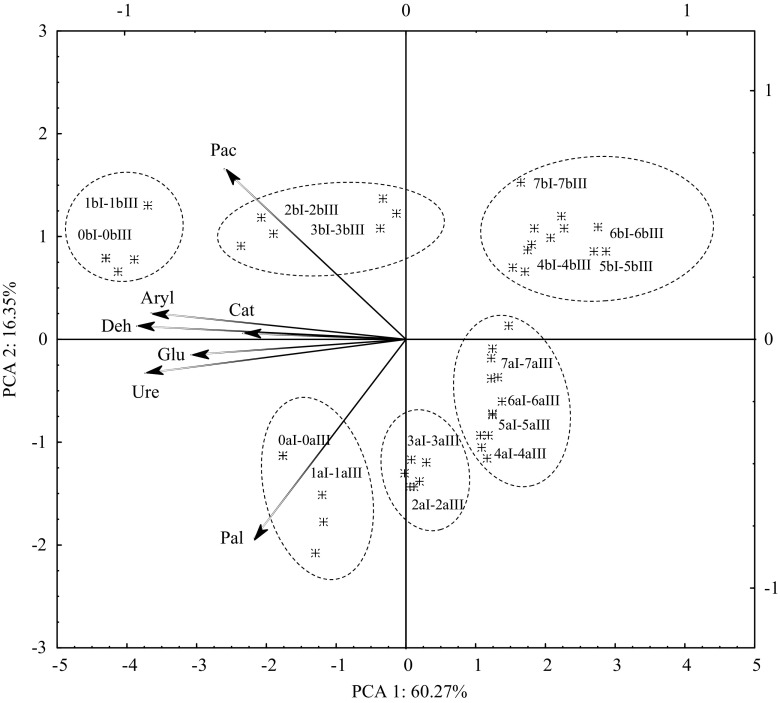
The enzymatic activity of soil contaminated with T + M + S, represented with the PCA. *Deh* dehydrogenases, *Cat* catalase, *Ure* urease, *Pac* acid phosphatase, *Pal* alkaline phosphatase, *Aryl* arylsulfatase, *Glu* β-glucosidase. Dose of herbicide in kilograms DM of soil: *0* 0 mg (control), *1* 0.6721 mg, *2* 13.442 mg, *3* 26.884 mg, *4* 53.768 mg, *5* 107.536 mg, *6* 215.072 mg, *7* 430.144 mg. Date of analysis (days): *a* 30 days, *b* 60 days; number of repetitions: *1*–*5*

The PCA inclusive of the persistence of the T + M + S mixture in the soil demonstrated some detailed and significant relationships (Fig. 3). Both after 30 and 60 days of the experiment, the distribution of vectors around the axis representing the first principal component, which described 60.27 % of the total variance of the data, points out that the activity of all the enzymes was negatively correlated with this variable, irrespective of a dose of the herbicide. The PCA results proved that the inhibition of the soil’s enzymatic activity was stronger on day 60 than on day 30, which was confirmed by the distribution of cases on the plane and their position relative to the vectors.

### The growth and development of maize

**Fig. 4:**
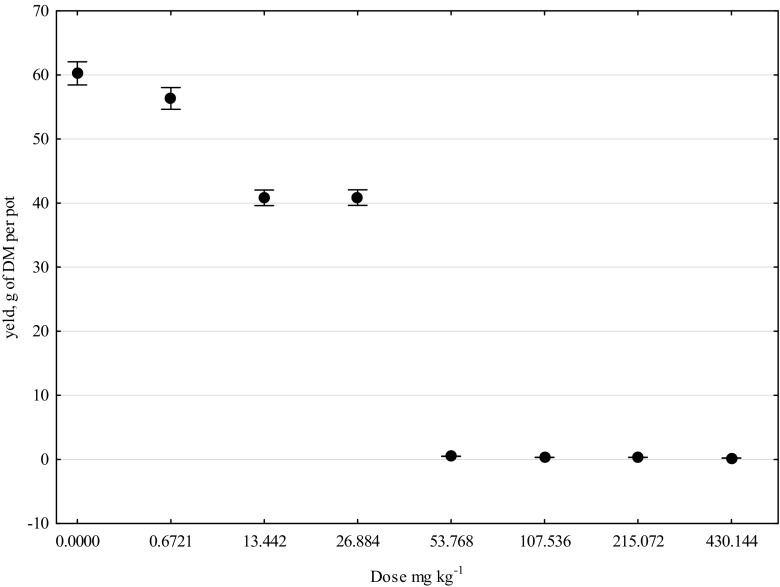
Effect of herbicide Lumax 537.5 SE (T + M + S) on yield of corn, grams of DM per pot

The key determinant of the phytotoxicity of the mixture of terbuthylazine, mesotrione, and S-metolachlor was the dosage of the herbicide (Fig. 4). It was demonstrated unquestionably that the above substances, if applied in the manufacturer-recommended dose, did not cause any irregularities in the growth and development of maize. However, when introduced to soil in excessive quantities, they contributed to a dramatic inhibition of the crop’s growth. The characteristic symptoms of maize’s biological processes being distorted by the stress caused by soil contamination with Lumax 537.5 SE were the deformation of the root system and chlorosis of the leaves. Doses of T + M + S above 53.768 mg kg^−1^ DM of soil were particularly toxic, having led to the necrosis of maize plants in the BBCH 13 phase.

## Discussion

### Soil microorganisms

The influence of herbicides on the microbiological and biochemical activity of soil cannot be described by simple relationships because pesticides are often composed of not one but two or three active ingredients. Such preparations are more toxic to the soil microbiome (Tejada 2009). This is what occurred in our study concerning the herbicide Lumax 537.5 SE, which inhibited the multiplication of oligotrophic, organotrophic, and *Azotobacter* spp. bacteria as well as actinomycetes and fungi, while stimulating the growth of endospore-forming oligotrophic bacteria. The microorganisms which were distinctly most sensitive to the tested herbicide were *Azotobacter* spp. bacteria. The sensitivity of these bacteria to pesticides was also reported by Milošević et al. (2004) and Elbashier et al. (2016). The varied response of microorganisms to tested pollution is associated with their succession (Pérez-Bárcena et al. 2014) induced by the death of sensitive microorganisms and reproduction of more tolerant ones (Crouzet et al. 2010; Kucharski et al. 2016). Changes in the multiplication of microorganisms in soil polluted with T + M + S which were observed in our experiment may have also been caused by the fact that the active substances of Lumax 537.5 SE undergo chemical and microbiological degradation at different rates and therefore affect the soil microbiome differently. The half-life of these substances varies from a few days to over a month (O’Connell et al. 1998), and one of them, i.e., metolachlor, is distinguished by its high mobility and solubility. The influence of herbicides containing more than one active substance is bound to be a complex issue. Nonetheless, even the most persistent substances in the soil environment can be metabolized by microorganisms. According to Arbeli and Fuentes (2007), the highest capability to degrade pesticides, including herbicides, is demonstrated by microorganisms of the genera *Arthrobacter*, *Pseudomonas*, *Bacillus*, *Mycoplana*, *Agrobacterium*, *Corynebacterium*, *Flavobacterium*, *Nocardia*, and *Trichoderma*. The biodegradation of Lumax 537.5 SE is actively participated by the microorganisms specified in Table 11. The biodegradation of pesticides in the soil environment is most often achieved by a consortium of microorganisms rather than single species (Castillo et al. 2006). This is the reason why autochthonous bacteria present in natural soil ecosystems play such an important role.

**Table 11:** Microorganisms which degrade the substances contained in the herbicide Lumax 537.5 SE

Substances	Microorganisms	References
Terbuthylazine	*Escherichia coli*, *Pseudomonas* sp., *Rhodococcus*, *Fusarium oxysporum*, *Aspergillus oryzae*, *Lentinula edodes* (shiitake mushroom), *Penicillium brevicompactum*, *Lecanicillum sakenae*	Martínez-Iñigo et al. 2010, Aranaz et al. 2008, Johannesen and Aamand 2003, Grenni et al. 2009, Pinto et al. 2012
Mesotrione	*Bacillus megaterium*, *Bacillus* sp., *Vibrio fischeri*, *Pantoea ananatis*, *Tetrahymena pyriformis*	Bardot et al. 2015, González et al. 2012, Bonnet et al. 2008, Pileggi et al. 2012
S-Metolachlor	*Bacillus simplex*, *Moraxella* sp., *Moraxella macacae*, *Moraxella bovis*, *Xanthobacter*, *Enterobacter hormaechei*, *Enterobacter cancerogenus*, *Enterobacter kobei*, *Enterobacter aerogenes*, *Enterobacter asburiae*, *Enterobacter amnigenus*, *Enterobacter nimipressuralis*, *Leclercia adecarboxylata*, *Tatumella ptyseos*, *Pantoea ananatis*, *Pantoea agglomerans*, *Pantoea stewartii*, *Kosakonia cowanii*, *Salmonella bongori*, *Kosakonia radicincitans*, *Kosakonia oryzae*, *Klebsiella variicola*, *Klebsiella pneumoniae*, *Pseudomonas alcaligenes*, *Candida xestobii*, *Beauveria bassiana*	Munoz et al. 2011, Villarreal et al. 1991, Martins et al. 2007, Kos and Celar 2013, Martins et al. 2011

The application of the mixture of terbuthylazine, mesotrione, and S-metolachlor contributed to the disturbance of the balance in the soil ecosystem, causing changes not only in the counts of microorganisms but also in the structure of soil-dwelling microbial communities and their diversity. The stability of the soil microbiome in our study was evaluated according to the colony development index (CD) of microorganisms (Sarathchandra et al. 1997) and the index of ecophysiological diversity (EP) of microorganisms (De Leij et al. 1993). Both of these indices are based on the concept of r- and K-strategists. These indicators allowed us to make observations of changes in the proportions of rapidly and slowly growing microorganisms because the genetic differences between microorganisms enable them to adjust to changes in the environment and to survive (De Leij et al. 1993; Borowik and Wyszkowska 2016).

The ecophysiological diversity (EP) index provides more information about the response of microorganisms to the soil contamination with the herbicide Lumax 537.5 SE. Having analyzed the values of this index, we can conclude that organotrophic bacteria and actinomycetes were characterized by the highest diversity. This in turn corresponds to the highest ecophysiological diversity. In our research, an excess amount of the M + T + S mixture significantly depressed the ED index value of fungi, which suggests that sensitive species were supplanted by more tolerant ones.

The various effects of the herbicide on the diversity of microorganisms observed in our experiment are not a unique phenomenon. Similar changes in the diversity of soil microorganisms were noticed by Lone et al. (2014), who tested soproturon, metribuzin, clodinafop propargyl, atlantis, and sulfosulfuron. Kucharski et al. (2016), having applied the herbicide Boreal 58 WG to soil, observed an increase in the CD index of organotrophic bacteria, actinomycetes, and fungi compared to their populations in the control soil. Baćmaga et al. (2015) also demonstrated that such active ingredients as diflufenican, mesosulfuron-methyl, and iodosulfuron-methyl-sodium modified values of the colony development (CD) and the ecophysiological diversity (EP) indices of organotrophic bacteria. In turn, a mixture of pethoxamide and terbuthylazine only slightly affected these parameters (Tomkiel et al. 2014).

### Soil enzymes

The soil pollution with the mixture of terbuthylazine, mesotrione, and S-metolachlor, by disturbing the metabolic profile of the soil, changed the activity of enzymes which participate in the transformations of carbon, nitrogen, phosphorus, and sulfur. A change in the biochemical properties of soil induced by excessively high doses of Lumax 537.5 SE was manifested by the indices measuring the effect of the herbicide on individual enzymes. The determination of these indices enabled us to state objectively whether the analyzed ecosystem was stable and able to sustain an appropriate balance. The coefficients of the impact of T + M + S mixture on the activity of soil enzymes in nearly all cases, irrespective of the date of soil sampling (the growth phase of maize), had negative values. They therefore pointed up the correlation between the growing disturbance of the soil’s homeostasis and the increasing inhibition caused by the tested preparation. Dehydrogenases were demonstrably sensitive to the excess M + T + S mixture, in contrast to alkaline phosphatase and acid phosphatase, which were the most tolerant. Dehydrogenases were also the most sensitive soil enzymes to the herbicide Apyros 75 WG (Kucharski and Wyszkowska 2008) and the mixture of diflufenican, mesosulfuron-methyl, and iodosulfuron-methyl-sodium (Baćmaga et al. 2015). Similar results were delivered by Lone et al. (2014), who tested six herbicides (soproturon, metribuzin, clodinafop propargyl, atlantis, and sulfosulfuron) and showed that dehydrogenases were most sensitive to the applied chemicals. In our study, catalase, urease, β-glucosidase, and arylsulfatase responded similarly to dehydrogenases to the tested application of Lumax 537.5 SE and therefore these enzymes can be seen as an additional indicator in an evaluation of soil pollution monitored by the activity of dehydrogenases. Alkaline and acid phosphatase proved to be rather unhelpful in this regard.

Many researchers (Martins et al. 2011; Nikoloff et al. 2013; Bello et al. 2013) share the opinion that a decreased enzymatic activity of soil is the response to a biotic stress induced by soil contamination with herbicides. Other studies as well (Sofo et al. 2012, Vlădoiu et al. 2015) implicate that herbicides, given certain circumstances, can act as strong inhibitors of enzymes. Tejada (2009) also showed that a mixture of glyphosate and diflufenican had a stronger inhibitory effect on the microbiological activity of soil than each of these substances applied separately. This impact was more evident in loamy sand than in sandy loam. Such observations lead to the conclusion that degradation of individual pesticides is strictly dependent not only on the duration of their presence in soil but also on the physicochemical properties of soil. The soil in our study was a sandy one of pH_KCl_ = 7.00 and carbon content of 7.05 g C kg^−1^ DM of soil. It was therefore the type of soil which creates very good conditions for the development of soil microbiota. This observation is important in that that some microorganisms were able to use the M + T + S mixture as a source of nutrients, which consequently may have affected the biosynthesis of enzymes via induction or repression of processes. Both pesticides and their metabolites, which often become more toxic than the original substances, can also affect the physiological process of microorganisms, e.g., the lysis of cells or modification of the cell membrane, and this can contribute to changes in the activity of soil enzymes (Floch et al. 2011, Singh and Goshal 2010). Persistent exposure of a given ecosystem to a variety of stimuli leads to the formation of adequate defense mechanisms, able to sustain an adequate biological equilibrium of soil (Griffiths and Philippot 2013).

To sum up our discussion on the effect of herbicide on the biological activity of soil, both our results and references (Bello et al. 2013; Kucharski et al. 2016) suggest that herbicides applied according to the guidelines of good agricultural practice either do not alter the enzymatic activity of soils or they cause transient fluctuations in the activity of some enzymes. A dramatic decrease in the enzymatic activity is noted in a soil environment which contains excessive quantities of herbicides.

### Growth and development of maize

The microbiological and enzymatic characteristics of soil are a reflection of its fertility, which in turn correlates with the volume and quality of yields. Lumax 537.5 SE has been in use in Poland since 2008. It is one of the most popular herbicides applied in maize fields. The active substances of this preparation did not produce a negative effect on the growth and development of maize when applied in the optimum dose. All the other doses (higher than recommended) were toxic to both weeds and maize. This finding proves that levels of herbicides as well as their side effect on crops and the soil environment should be monitored constantly, especially when weed eradication is intensive.

Our results concerning the impact of the M + T + S mixture on plants correspond to the results obtained by Bettiol et al. (2016), who assessed the phytotoxic effect of three herbicides (chloroxynil, bromoxynil, and ioxynil) on the germination of seeds and elongation of roots of *Leptidium sativum*, finding out that ioxynil was the most toxic preparation. Wyszkowska (2002), who tested trifluarin, the active ingredient of the herbicide Treflan 480 EC, showed the negative effect of this chemical compound on the growth of spring oilseed rape and white mustard. These plants also responded negatively, by producing lower yields, to the excessive amount of the herbicide Triflurotox 250 EC (Wyszkowska and Kucharski 2004). Kucharski and Wyszkowska (2008) demonstrated an inhibitory effect of Apyros 74 WG on the growth of oat. Baćmaga et al. (2014) reported a negative influence of a mixture of diflufenican, mesosulfuron-methyl, and iodosulfuron-methyl-sodium on spring wheat, while Elbashier et al. (2016) obtained such results testing the effect of Sevin on carrot. Based on our results as well as references (Schmalenberger and Tebbe 2002; Bettiol et al. 2016), it can be concluded that when excessive amounts of herbicides enter the soil environment they disturb the proper growth and development of crops. The symptoms observed under such conditions include retardation of the growth; delayed flowering; undeveloped leaves; chlorosis; blanching; browning or redding of leaves; their crispation, curling, and wilting; and consequently, the necrosis of plants.

## Conclusions

Herbicides can be toxic not only to the weeds that they are intended to eradicate but also to crops. When permeating into soil, herbicides can pose a threat to soil-borne organisms and to plants. This study describes the influence of a mixture of three active substances: terbuthylazine, mesotrione, and S-metolachlor contained in the herbicide Lumax 537.5 SE, on soil microorganisms, soil enzymes, and maize. It was demonstrated that the stress induced by these chemical compounds led to changes in the values of the colony development (CD) indices of organotrophic bacteria, actinomycetes, and fungi and ecophysiological diversity (EP) indices of fungi. Changes in the ecophysiological diversity index of organotrophic bacteria and actinomycetes were small.

A mixture of these chemical compounds was also a strong inhibitor of dehydrogenases, to a less degree urease, β-glucosidase, catalase, and arylsulfatase, and a weak inhibitor of phosphatase. Excessive amounts of the herbicide Lumax 537.5 SE in soil inhibited the growth and development of maize. The results prove unquestionably that the tested herbicide should be applied strictly in line with the application regime, including its dosage. When the application of the herbicide respected the manufacturer’s recommendations, the preparation did not cause any larger disturbances in the soil’s homeostasis. However, its excessive doses (from 13.442 to 430.144 mg kg^−1^ DM of soil) proved to be dangerous. The results presented above confirm that a combination of microbiological and biochemical properties with a simultaneous determination of the response of crops enables a complex assessment of the quality of soil exposed to herbicides.

## References

[CR1] Alef K, Nannipieri P, Alef K, Nannipieri P (1998). Methods in applied soil microbiology and biochemistry.

[CR2] Alexander M (1973). Microorganisms and chemical pollution. Bioscience.

[CR3] Aranaz A, Gibello A, Álvarez J, Mata AI, Rodríguez A, Fallola C, Fernández-Garayzábal JF, Domínguez L (2008). Mycobacterium peregrinum infection in farmed European tench (*Tinca tinca* L). Vet Microbiol.

[CR4] Arbeli Z, Fuentes CL (2007). Accelerated biodegradation of pesticides: an overview of the phenomenon, its basis and possible solutions and a discussion on the tropical dimension. Crop Protec.

[CR5] Baćmaga M, Kucharski J, Wyszkowska J, Borowik A, Tomkiel M (2014). Responses of microorganisms and enzymes to soil contamination with metazachlor. Environ Earth Sci.

[CR6] Baćmaga M, Borowik A, Kucharski J, Tomkiel M, Wyszkowska J (2015). Microbial and enzymatic activity of soil contaminated with a mixture of diflufenican + mesosulfuron-methyl + iodosulfuron-methyl-sodium. Environ Sci Pollut Res.

[CR7] Bardot C, Besse-Hoggan P, Carles L, Le Gall M, Clary G, Chafey P, Federici C, Broussard C, Batisson I (2015). How the edaphic *Bacillus megaterium* strain Mes11 adapts its metabolism to the herbicide mesotrione pressure. Environ Pollut.

[CR8] Bello D, Trasar-Cepeda C, Leirós MC, Gil-Sotres F (2013). Modification of enzymatic activity in soils of contrasting pH contaminated with 2,4-dichlorophenol and 2,4,5-trichlorophenol. Soil Biol Biochem.

[CR9] Bettiol C, De Vettori S, Minervini G, Zuccon E, Marchetto D, Ghirardini AV, Argese E (2016). Assessment of phenolic herbicide toxicity and mode of action by different assays. Environ Sci Pollut Res.

[CR10] Blanchoud H, Moreau-Guigon E, Farrugia F, Chevreuil M, Mouchel JM (2007). Contribution by urban and agricultural pesticide uses to water contamination at the scale of the Marne watershed. Sci Total Environ.

[CR11] Bonnet JL, Bonnemoy F, Dusser M, Bohatier J (2008). Toxicity assessment of the herbicides sulcotrione and mesotrione toward two reference environmentalmicroorganisms: *Tetrahymena pyriformis* and Vibrio fischeri. Arch Environ Contam Toxicol.

[CR12] Borowik A, Wyszkowska J (2016). Impact of temperature on the biological properties of soil. Int Agrophys.

[CR13] Bro E, Devillers J, Millot F, Decors A (2016). Residues of plant protection products in grey partridge eggs in French cereal ecosystems. Environ Sci Pollut Res.

[CR14] CASAFE (2011 vs 2012) Mercado argentino de productos fitosanitarios, argentine market of plant protection products. http://www.casafe.org/biblioteca/estadisticas/

[CR15] Castillo MA, Felisa N, Aragón P, Cuesta G, Sabater C (2006). Biodegradation of the herbicide diuron by streptomycetes isolated from soil. Int Biodeterior Biodegrad.

[CR16] Clark GM, Goolsby DA (2000). Occurrence and load of selected herbicides and metabolites in the lower Mississippi river. Sci Total Environ.

[CR17] Crouzet O, Batisson I, Besse-Hoggan P, Bonnemoy F, Bardot C, Poly F, Bohatier J, Mallet C (2010). Response of soil microbial communities to the herbicide mesotrione: a dose-effect microcosm approach. Soil Biol Biochem.

[CR18] De Leij FAAM, Whipps JM, Lynch JM (1993). The use of colony development for the characterization of bacterial communities in soil and on roots. Microb Ecol.

[CR19] Delgado-Moreno L, Peña A (2009). Compost and vermicompost of olive cake to bioremediate triazines-contaminated soil. Sci Total Environ.

[CR20] Diez C, Barrado E (2010). Soil-dissipation kinetics of twelve herbicides used on a rain-fed barley crop in Spain. Anal Bioanal Chem.

[CR21] Directive 2009/128/EC of the European Parliament and of the Council establishing a framework for community action to achieve the sustainable use of pesticides, UE (2009) L 309. http://www.audace-ass.com/news_database/amending_dir_91_414/EU-Parliament/2009-11-24-dir/Directive_2009-128-EC_European-Parliamentd_Council_21Oct2009_sustainable-use-PPP_PL_24Nov2009.pdf

[CR22] Elbashier MMA, Shao XM, Mohmmed A, Ali AAS, Osman BH (2016). Effect of pesticide residues (Sevin) on carrot (*Daucus carota* L.) and free nitrogen fixers (*Azotobacter* spp.). Agricultural Science.

[CR23] EPPO 2016 Guidelines of the European and Mediterrancean Plant Protection Organization (EPPO) concerning research on the efficiency of plant protection chemicals. Ministry of Agriculture and Rural Development (13.03.2016)

[CR24] Fenglerowa W (1965). Simple method for counting *Azotobacter* in soil samples. Acta Microbiol Pol.

[CR25] Floch C, Chevremont AC, Joanico K, Capowiez Y, Criquet S (2011). Indicators of pesticide contamination: soil enzyme compared to functional diversity of bacterial communities via Biolog Ecoplates. Eur J Soil Biol.

[CR26] González S, López-Roldán R, Cortina JL (2012). Presence and biological effects of emerging contaminants in Llobregat River basin: a review. Environ Pollut.

[CR27] Grenni P, Gibello A, Caracciolo AB, Fajardo C, Nande M, Vargas R, Saccà ML, Martinez-Iñigo MJ, Ciccoli R, Martín M (2009). A new fluorescent oligonucleotide probe for *in situ* detection of *s*-triazine-degrading *Rhodococcus wratislaviensis* in contaminated groundwater and soil samples. Water Res.

[CR28] Griffiths BS, Philippot L (2013). Insights into the resistance and resilience of the soil microbial community. FEMS Microbiol Rev.

[CR29] Herbicide Resistance Action Committee HRAC (2016) http://www.hracglobal.com/

[CR30] Idziak R, Woźnica Z (2008). Efficacy of herbicide Callisto 100 sc applied with adjuvants and a mineral fertilizer. Acta Agroph.

[CR31] ISO 10390 (2005) Soil quality—determination of pH

[CR32] ISO 11261 (1995). Soil quality—determination of total nitrogen—modified Kjeldahl method.

[CR33] IUSS Working Group WRB: World Reference Base for Soil Resources (2014) International soil classification system for naming soils and creating legends for soil maps. WRB 106:182 ss

[CR34] Jastrzębska E, Kucharski J (2007). Dehydrogenases, urease and phosphatases activities of soil contaminated with fungicides. Plant Soil Environ.

[CR35] Johannesen H, Aamand J (2003). Mineralization of aged atrazine, terbuthylazine, 2,4-D, and mecoprop in soil and aquifer sediment. Environ Toxicol Chem.

[CR36] Jones DL, Edwards-Jones G, Murphy DV (2011). Biochar mediated alterations in herbicide breakdown and leaching in soil. Soil Biol Biochem.

[CR37] Kaczmarek S, Matysiak K, Kierzek R (2012). Weed control efficacy and selectivity of preemergence herbicides in *Sorghum vulgare* Perz. cultivation. Prog Plant Prot.

[CR38] Klute A (1996) Methods of soil analysis. Part 1: physical and mineralogical methods. American Society of Agronomy. Inc., Madison, pp 1188

[CR39] Kos K, Celar F (2013). Sensitivity of the entomopathogenic fungus Beauveria bassiana (Bals.-Criv.) Vuill. to selected herbicides. Pest Manag Sci.

[CR40] Kucharski J, Wyszkowska J (2008). Biological properties of soil contaminated with the herbicide Apyros 75 WG. J Elem.

[CR41] Kucharski J, Tomkiel M, Baćmaga M, Borowik A, Wyszkowska J (2016). Enzyme activity and microorganisms diversity in soil contaminated with the boreal 58 WG herbicide. J Environ Sci Health B.

[CR42] Lone AH, Raverkar KP, Pareek N, Chandra R (2014). Response of soil microbial communities to the selective herbicides: a microcosm approach. JPAM.

[CR43] Long YH, Li RT, Wu XM (2014). Degradation of S-metolachlor in soil as affected by environmental factors. J Soil Sci Plant Nutr.

[CR44] Martin J (1950). Use of acid rose bengal and streptomycin in the plate method for estimating soil fungi. Soil Sci.

[CR45] Martínez-Iñigo MJ, Gibello A, Lobo C, Nande M, Vargas R, Garbi C, Munoz A, Koskinen WC, Cox L, Sadowsky ZJ (2010). Biodegradation and mineralization of metolachlor and alachlor by Candida xestobii. J Agric Food Chem.

[CR46] Martins PF, Martinez CO, de Carvalho G, Carneiro PIB, Azevedo RA, Pileggi SAV, de Melo IS, Pileggi M (2007). Selection of microorganisms degrading S-metolachlor herbicide. Braz Arch Biol Techn.

[CR47] Martins PF, Carvalho G, Gratão LG, Dourado MN, Pileggi M, Araújo WL, Azevedo RA (2011). Effects of the herbicides acetochlor and metolachlor on antioxidant enzymes in soil bacteria. Process Biochem.

[CR48] Milošević N, Govedarica M, Cvijanović G (2004). Microorganisms as indicators of herbicide effect on biological activity of soil. Acta Herbol.

[CR49] Munoz A, Koskinen WC, Cox L, Sadowsky MJ (2011). Biodegradation and mineralization of metolachlor and alachlor by *Candida xestobii*. J Agric Food Chem.

[CR50] Navarro S, Bermejo S, Vela N, Hernández J (2009). Rate of loss of simazine, terbuthylazine, isoproturon, and methabenzthiazuron during soil solarization. J Agric Food Chem.

[CR51] Nelson DW, Sommers LE (1996) Total carbon, organic carbon, and organic matter. Method of soil analysis: chemical methods. In: D.L. Sparks (Ed) American Society of Agronomy, Madison, WI. (pp. 1201–1229)

[CR52] Nikoloff N, Escobar L, Soloneski S, Larramendy ML (2013). Comparative study of cytotoxic and genotoxic effects induced by herbicide S-metolachlor and its commercial formulation Twin Pack Gold® in human hepatoma (HepG2) cells. Food Chem Toxicol.

[CR53] O’Connell PJ, Harms TH, Allen JRF (1998). Metolachlor, S-metolachlor and their role within sustainable weed-management. Crop Prot.

[CR54] Öhlinger R, Schinner F, Ohlinger R, Kandler E, Margesin R (1996). Dehydrogenase activity with the substrate TTC. Methods in soil biology.

[CR55] Onta H, Hattori T (1983). Oligotrophic bacteria on organic debris and plant roots in paddy field. Soil Biol Biochem.

[CR56] Parkinson D, Gray FRG, Williams ST (1971). Methods for studying the ecology of soil micro-organism.

[CR57] Peña D, López-Piñeiro A, Albarrán A, Becerra D, Sánchez-Llerena J (2015). Environmental fate of the herbicide MCPA in agricultural soils amended with fresh and aged de-oiled two-phase olive mill waste. Environ Sci Pollut Res.

[CR58] Pérez-Bárcena JF, Ahuatzi-Chacón D, Castillo-Martínez KL, Ruiz-Ordaz N, Galíndez-Mayer J, Juárez-Ramírez C, Ramos-Monroy O (2014). Effect of herbicide adjuvants on the biodegradation rate of the methylthiotriazine herbicide prometryn. Biodegradation.

[CR59] Pileggi M, Pileggi SA, Olchanheski LR, da Silva PA, Munoz Gonzalez AM, Koskinen WC, Barber B, Sadowsky MJ (2012). Isolation of mesotrione-degrading bacteria from aquatic environments in Brazil. Chemosphere.

[CR60] Pinto AP, Serranoa C, Piresa T, Mestrinhoa E, Diasa L, Teixeiraa DM, Caldeira AT (2012). Degradation of terbuthylazine, difenoconazole and pendimethalin pesticides by selected fungi cultures. Sci Total Environ.

[CR61] Pose-Juan E, Sánchez-Martín MJ, Herrero-Hernández E, Rodríguez-Cruz MS (2015). Application of mesotrione at different doses in an amended soil: dissipation and effect on the soil microbial biomass and activity. Sci Total Environ.

[CR62] Sarathchandra SU, Burch G, Cox NR (1997). Growth patterns of bacterial communities in the rhizoplane and rhizosphere of with clover (*Trifolium repens* L.) and perennial ryegrass (*Lolium perenne* L.) in long-term pasture. Appl Soil Ecol.

[CR63] Schmalenberger A, Tebbe CC (2002). Bacterial community composition in the rhizosphere of a transgenic, herbicide-resistant maize (*Zea mays*) and comparison to its non-transgenic cultivar Bosphore. FEMS Microbiol Ecol.

[CR64] Siles JA, Rachid CTCC, Sampedro I, García-Romera I, Tiedje JM (2014). Microbial diversity of a Mediterranean soil and its changes after biotransformed dry olive residue amendment. PLoS One.

[CR65] Singh P, Goshal N (2010). Variation in total biological productivity and soil microbial biomass in rainfed agroecosystems: impact of application of herbicide and soil amendments. Agric Ecosyst Environ.

[CR66] Snarska K, Konecki R (2010). Assessment of effectiveness of selected herbicides used for limiting weeds in sorghum. Prog Plant Prot.

[CR67] Sofo A, Scopa A, Dumontet S, Mazzatura A, Pasquale V (2012). Toxic effects of four sulphonylureas herbicides on soil microbial biomass. J Environ Sci Health Part B.

[CR68] Soltani N, Shropshire C, Sikkema PH (2006). Responses of winter wheat (*Triticum aestivum* L.) to autumn applied post-emergence herbicides. Crop Prot.

[CR69] Statsoft, Inc., Statistica (2015) Data analysis software system, version 12.0. http://www.statsoft.com

[CR70] Tandon S, Pujari A, Sand NK (2012). Degradation of Fentrazamide herbicide in soil under aerobic condition. Bull Environ Contam Toxicol.

[CR71] Tejada M (2009). Evolution of soil biological properties after addition of glyphosate, diflufenican and glyphosate+diflufenican herbicides. Chemosphere.

[CR72] Tomkiel M, Wyszkowska J, Kucharski J, Baćmaga M, Borowik A (2014). Response of micoorganisms and enzymes to soil contamination with the herbicide successor T 550 SE. Environ Prot Eng.

[CR73] Villarreal DT, Turco RF, Konopka A (1991). Propachlor degradation by a soil bacterial community. Appl Environ Microbiol.

[CR74] Vlădoiu DL, Filimon MN, Ostafe V, Isvoran A (2015). Effects of herbicides and fungicides on the soil chitinolytic activity. A molecular docking approach. Ecol Chem Eng S.

[CR75] Włodarczyk M (2014). Influence of formulation on mobility of metazachlor in soil. Environ Monit Assess.

[CR76] Wyszkowska J (2002). Effect of soil contamination with Treflan 480 EC on biochemical properties of soil. Pol J Environ Stud.

[CR77] Wyszkowska J, Kucharski J (2004). Biochemical and physicochemical properties of soil contaminated with herbicide Triflurotox 250 EC. Pol J Environ Stud.

